# Balance and deep connections: the impact of physical activity on body and sexual self-esteem, psychological distress, and marital satisfaction among adults

**DOI:** 10.3389/fspor.2024.1343951

**Published:** 2024-04-11

**Authors:** Haifa Snani, Hela Snani, Amayra Tannoubi, Oussama Gaied Chortane, Fairouz Azaiez

**Affiliations:** ^1^ Department of Education, Higher Institute of Sport, and Physical Education of Sfax, University of Sfax, Sfax, Tunisia; ^2^ Higher Institute of Sport and Physical Education of El Kef, University of Jendouba, Jendouba, Tunisia; ^3^ Higher Institute of Sport and Physical Education of Ksar-Said, Universite de La Manouba, Tunis, Tunisia; ^4^ Department of Education, Higher Institute of Sport, and Physical Education of Gafsa, University of Gafsa, Gafsa, Tunisia

**Keywords:** sexual self-esteem, body self-esteem, psychological distress, marital satisfaction, engagement in physical activities

## Abstract

To date, no research has examined the relationship between sexual self-esteem, body self-esteem, psychological distress, marital satisfaction, and engagement in physical activities. Through a study involving 443 adults the aim of this research was to analyze the connections between engaging in physical activities, body self-esteem, sexual self-esteem, marital satisfaction, and psychological distress among adults. The results obtained through Partial Least Squares (PLS) modeling with SmartPLS reveal significant interconnections among these variables. The correlation between body self-esteem and psychological distress, the positive relationship between sexual satisfaction and positive sexual self-esteem, as well as the beneficial influence of engaging in physical activities on marital satisfaction, are key highlights of this study. The robustness of the measures, confirmed by high coefficients, strengthens the credibility of the results. Despite these advancements, the cross-sectional nature of the study emphasizes the need for longitudinal research to gain a deeper understanding of psychological and relational dynamics over time. In conclusion, this study offers significant contributions to promoting balanced marital relationships, highlighting the importance of considering body and sexual self-esteem, psychological distress, and physical activity within the context of human relationships.

## 1 Introduction

The term “Physical or Sports Activities” (APS) includes various practices. They can be sporting, competitive, recreational, or extreme. They may also be part of a lifestyle, including daily activities. These activities involve using the body, whether done alone or in groups. The significance of Physical or Sports Activities for overall health is undeniable. It goes beyond just being free of disease or infirmity. It encompasses complete well-being on physical, mental, and social levels (1).

Participation in Physical or Sports Activities throughout life is vital for an individual's physical health (2). Exercise indeed provides numerous health benefits, such as weight control, reduced risk of diabetes, cancer, and osteoporosis, as well as stress reduction (3). Moreover, physical activity is considered a key factor in “maintaining, improving, or even recovering health” (4). The psychological benefits of physical exercise are manifold: enhancing self-confidence, assertion, and better self-image perception (5). Furthermore, sports facilitate the healthy release of aggression (6), the realization of potential, and the pursuit of specific goals, while also minimizing the prevalence of emotional and social disorders (2), serving as a means to regain personal physical and mental balance (6, 7).

According to Martinsen and Morgan (8), the relationship between regular physical activity and psychological well-being has been well established. Exercise is indeed negatively associated with high scores of depression (8, 9) and trait anxiety (3), while being positively linked to indices of mental health such as psychological well-being (10). Some even view physical activity as a form of psychotherapy (6).

Additionally, Biddle (11) suggests that moderate-intensity physical activity is moderately correlated with increased vigor, as well as decreased tension, fatigue, and confusion, and weakly correlated with decreased anger.

Weinberg and Gould (12) have associated physical activity with decreased stress and hostility, as well as increased emotional stability and sexual satisfaction. On the other hand, several studies have linked physical activity to various subjective well-being variables (13). For instance, McAuley et al. (14) demonstrated that self-efficacy increases when people succeed in their involvement in physical activity, positively influencing subjective well-being. Moreover, the authors argue that satisfaction with physical functioning and appearance is directly associated with various components of subjective well-being (life satisfaction, positive and negative affects) in middle-aged and older adults (15).

Furthermore, physical activity is associated with positive self-esteem (16). Sonstroem, defined as our perception of self-worth and how others value us. Self-esteem plays a crucial role in feeling good about us and being comfortable in our skin. It significantly influences mental health, behavior, and perceptions of us as sexual partners, making it a key indicator of psychological well-being (17).

Additionally, research indicates that physical activity improves body image and self-empowerment (18). Body image, which encompasses an individual's feelings, attitudes, memories, and experiences related to their body (19) is essential for building self-esteem. Sports psychology emphasizes the role of body perception in self-esteem construction. Engaging in physical activity is linked to improved body image, reducing dissatisfaction with one's physical appearance (9).

Thus, McAuley and Rudolph (10) conclude that there is sufficient evidence supporting a positive association between physical activity and subjective well-being, both in adults and older individuals. Moreover, studies also suggest that psychological factors such as self-esteem, body image, and depression affect sexual satisfaction in both men and women. Researchers have identified overall self-esteem as an essential component of a healthy sexual functioning (20). Similarly, Maslow (21) concluded that self-esteem, more than the level of sexual desire determines sexual behaviors and attitudes. The concept of sexual self-esteem is largely developed from models of overall self-esteem. Although understudied until today, it currently constitutes the best scientifically validated indicator for evaluating sexual thoughts and is defined as “positive evaluations of one's own sexuality, including evaluations of sexual thoughts, feelings, and behaviors, as well as perceptions of one's body in the sexual context (22). Good sexual self-esteem is crucial for sexual well-being and extends beyond sexual aspects to impact overall health. Low sexual self-esteem not only affects sexual life quality but can also impede sexological therapy. Moreover, if good sexual self-esteem is an essential component of sexual well-being, it also extends beyond the sexual sphere. Indeed, while good sexual self-esteem prevents potential sexological problems, it has other impacts on health. Clinically, the results of Ménard and Offinan (23) suggest that sexual satisfaction could be improved by interventions targeting sexual self-esteem, supporting the importance of specifically targeting these variables. Sexual satisfaction is, inherent to human sexuality and is a human right (24). It is also a key component in quality, of life, particularly in better physical and psychological health and overall well-being (25, 26). Several authors, such as Sprecher and Cate (27) even suggest that satisfactory sexuality contributes to overall life satisfaction, and vice versa. On their part, Auton-Cuff et al. (28) present several factors that facilitate and limit sexual self-esteem. One of them concerns the relationship that women have with their bodies, which would be particularly influential in establishing sexual self-esteem. However, very few studies focus on self-esteem in men. Moreover, numerous studies report a positive association between sexual satisfaction and marital satisfaction (29–35). This becomes a means to assess the strength and success of the relationship (36, 37). According to Bradbury et al. (38) marital satisfaction corresponds to the personal evaluation of the satisfaction felt by partners within their relationship. Conversely, partners’ sexual dissatisfaction is a reliable indicator of the high prevalence of marital distress, particularly in women (32).

Nevertheless, longitudinal studies report that fluctuations in sexual satisfaction are linked to fluctuations in marital satisfaction (34, 39). Like sexual satisfaction, marital satisfaction is related to self-esteem (40) concludes that self-esteem provides a good indication of the future marital satisfaction of partners. Additionally (41), demonstrates a relationship between positive self-perception and marital satisfaction. Research indicates a positive association between body image and marital satisfaction. A more fulfilling marital relationship tends to correspond with lower dissatisfaction concerning partners' bodies, and conversely (42). Furthermore, psychological distress can influence marital satisfaction and vice versa.

Studies have consistently shown that individuals facing higher levels of psychological distress tend to report lower quality in their marital relationships (43–45).

Kessler et al. (45) found that spouses experiencing psychiatric disorders such as anxiety or mood disorders reported higher levels of marital dissatisfaction compared to those without these conditions. Similarly, individuals experiencing unsatisfactory marital relationships exhibited twice the amount of psychological distress compared to those satisfied in their marriages. Moreover, individuals in fulfilling marriages displayed twice the level of sexual desire, sexual satisfaction, and sexual functioning (46). Therefore, it seems pertinent to investigate whether the psychological benefits of physical exercise in one spouse act as a protective factor on the marital dyad, considering that marital satisfaction is linked to individual well-being (47).

Numerous studies have demonstrated the various psychological and physical benefits associated with engaging in physical and sports activities. However, there appears to be a gap in research regarding the relationship between physical activity, body self-esteem, and their potential influence on self-esteem, sexual satisfaction, and marital satisfaction among adults, particularly during periods marked by reduced sports participation.

### 1.1 Study hypotheses

This study is based on several key hypotheses:

H1: We hypothesize that there is a positive relationship between body self-esteem and sexual self-esteem. This suggests that maintaining a positive body image correlates with higher satisfaction in sexual life.

H2: We postulate that there is a positive relationship between sexual self-esteem and marital satisfaction. This hypothesis suggests that marital satisfaction is correlated with positive sexual self-esteem.

H3: We propose that there is a correlation between marital satisfaction and body image.

H4: We hypothesize that there is a negative correlation between psychological distress and marital satisfaction. This suggests that higher levels of psychological distress are inversely proportional to marital satisfaction.

H5: We suggest that there is a positive relationship between regular physical activity and marital satisfaction. This hypothesis posits that consistent engagement in physical activity may lead to increased marital satisfaction. This proposition is supported by evidence indicating a likely connection between physical activity and marital contentment, as well as the known psychological benefits of exercise.

## 2 Materials and methods

### 2.1 Participants and procedure

Our study targeted adult men and women. The research was conducted in most Tunisian cities, starting with deploying a questionnaire on the Google Drive platform. Carried out online from April 21, 2021, to November 11, 2021, the survey was also shared across various social media platforms. Among the initial 520 received responses, 443 were retained after eliminating incomplete or aberrant information-containing questionnaires. Consequently, our study cohort comprised 443 participants, with an average age of 48 years.

Before answering the questionnaire, each participant provided informed consent to participate in the study. The entire research was approved by the Ethics Committee of the Faculty of Medicine of Sousse on March 21, 2022, with reference number No. 113 (Ref: CEFMS 113/2022).

Our study population consisted of 443 participants, predominantly female (52.4%), resulting in a sex ratio of 0.91. The average age of the participants was 42.84 ± 8.09 years, ranging from 24 to 77 years old, with 57.1% of participants being over 40 years old. Nearly all participants (*n* = 427) were married, with marriage duration ranging from 1 to 45 years and an average of 13.22 ± 8.09 years; 74.7% had at least 2 children.

### 2.2 Measures

Physical Activity Level: The assessment of physical activity level was conducted using the Ricci and GAGNON questionnaire, modified by Laureyns and Séné. This questionnaire determined a score ranging from 18 to 35 points. It comprised nine questions rated from 1 to 5 points, evaluating domestic, leisure, work-related activities, as well as sedentary behavior. Participants were classified into three groups based on their scores (Less than 18: Inactive, Between 18 and 35: Active, more than 35: Very Active) (48).

Body Self-Esteem was measured using the French version of the Body Esteem Scale, a multidimensional self-questionnaire. It consisted of 23 items rated on a five-point Likert scale, distributed into three subscales: “Appearance,” “Attribution,” and “Weight.” Higher scores indicated a better body esteem and a positive appreciation of appearance. Some items were reverse scored (4, 7, 9, 11, 13, 17, 18, 19, and 21). Various validation studies (49–53). Demonstrated a satisfactory internal consistency across the three subscales. The scale was validated with adolescents and young adults. The test-retest reliability (*n* = 151) for the “satisfaction with weight” subscale was 0.76; 0.69 for the “satisfaction with general appearance” subscale, and 0.63 for the “desire for change and negative effects associated with general appearance” subscale. Cronbach's alphas for the three subscales from the exploratory factor analysis of the BES (satisfaction with weight; satisfaction with general appearance; desire for change and affects associated with general appearance) were 0.90, 0.83, and 0.80, respectively (53).

Sexual Self-Esteem was assessed using a subscale of the Multidimensional Sexuality Inventory (54). IMS: Snell et al. translated by Ravart et al. (52). The IMS evaluated twelve spheres of sexuality using 60 Likert-type items on a five-point scale ranging from 1 “does not characterize me at all” to 5 “characterizes me a lot.” The authors reported good internal consistency of the subscale, with a Cronbach's alpha of 0.87, and good factorial validity of the IMS (52). Summing up the items of this subscale yielded a sexual self-esteem score. A higher score indicated better sexual self-esteem.

Psychological distress was measured using the Kessler Scale (K10) developed by Kessler and colleagues (45). According to the authors' conceptualization, psychological distress is a non-specific psychological state characterized by feelings reflecting depressive or anxious mood. A French translation of this instrument used in the Canadian Community Health Survey (CCHS) was employed in this study. Instructions highlighted indicating how often the participant felt, for example, sad or depressed in the last 30 days. Five response choices were offered: never, rarely, sometimes, most of the time, and all the time, associated with scores ranging from 0 to 4. Therefore, obtained results varied between 0 and 40, with a higher score indicating greater psychological distress.

Marital Satisfaction Level was measured using the Dyadic Adjustment Scale (DAS-4), developed by Sabourin, Valois, and Lussier (55). This instrument is the abbreviated French version of the Dyadic Adjustment Scale developed by Spanier (1976) (DAS-32), shortened to 4 items (DAS-4) (55). These 4 items were: (1) thinking about separation or divorce; (2) trusting the partner; (3) being happy in the relationship; (4) sharing positive elements. The DAS-4 underwent five studies with 8,256 married or dating participants. It proved its ability to measure the unidimensional aspect of marital satisfaction and predict couple stability while avoiding social desirability bias. Apart from being quick to complete, this scale is an effective measure for predicting couple dissolution (55). Each question was evaluated using a 5 or 6-point Likert scale depending on the different items. The sum of the four items constituted the satisfaction score. A high score indicated increased marital satisfaction. A score below 13 indicated a distressed spouse. It had good internal consistency, with a Cronbach's alpha of 0.89 for women and 0.78 for men (55).

The data analysis relies on using the PLS-SEM (Partial Least Squares Structural Equation Modeling) method with the software *SmartPLS 3.3.2.* This approach was chosen to assess the complex relationships between observable variables and latent variables, implementing Principal Component Analysis (PCA) to describe these associations. During model estimation, the PLS approach employs least squares regression methods. The goal of PLS modeling is to maximize the explained variance of the dependent latent variable. For instance, in modeling, it's possible to obtain empirical measures that highlight the correlations between indicators and constructs, as well as interactions between latent components (56, 57). Therefore, our aim is to explore theoretically established links based on field-collected data. In this perspective, empirical measures enable us to assess how well the theory aligns with data acquired through experience. The PLS method offers us the opportunity to evaluate the predictive capability of the model and determine its overall quality (56, 57).

## 3 Results

### 3.1 SMARTPLS model

The SmartPLS program was utilized for model estimation (v. 3.3.2). Using the PLS approach, it enables the description of complex associations between observable variables and latent variables through a statistical method known as Principal Component Analysis (PCA).

In our study, we conducted tests on both the measurement model and the structural model. These two sub-models are used together to compute the structural equations (56, 58). Utilizing the measurement model allows us to determine the nature of the relationships between observable indicators and latent variables. As for the structural model, its role is to identify causal links between latent variables when studying them. The primary objective is to establish whether certain latent variables influence others in the model, either directly or indirectly (59).

Following the PLS methodology, start by testing measurement and structural models. Then, check the reflexive measures of the dependent variable. Examine model hypotheses next, using the dependent variable formatively. This helps evaluate impacts on components and the overall effect. The measurement model assessment focuses on reliability, composite reliability, convergent, and discriminant validity.

### 3.2 Reliability and composite reliability for internal consistency

In a model constructed using SmartPLS, to assess construct reliability, we estimate it using three coefficients: Cronbach's alpha coefficient, composite reliability (CR) with Joreskog's rho, and Rho-A coefficient (Dijkstra-Henseler's Rho).

For acceptable internal consistency analysis, it's recommended that the Cronbach's alpha reliability coefficient should be above 0.6. The Joreskog's rho composite reliability index should be higher than 0.7 (60). The Rho-A reliability coefficient (61) falls between Cronbach's alpha and composite reliability. It's generally considered a good indicator for reliability assessment, with a recommended threshold of 0.70 (59).

Table 1 shows that the Cronbach's alpha coefficient ranges between 0.844 and 0.951. These values suggest that the indicators can consistently measure the constructs. Additionally, the values for Rho-A composite reliability were all adequate, ranging from 0.844 for marital satisfaction to 0.959 for the Distress construct (see Table 1).

**Table 1:** Convergent validity and reliability.

	Cronbach's alpha	Rho_A	Composite reliability	Average variance extracted (AVE)
Body appearance	0.951	0.954	0.958	0.696
Psychological distress	0.958	0.959	0.963	0.725
Physical activity	0.863	0.865	0.907	0.709
Marital satisfaction	0.844	0.844	0.906	0.762
Sexual self-esteem	0.942	0.943	0.956	0.811

### 3.3 Convergent validity

Convergent validity of indicators provides information on the extent to which they measure the constructs they are supposed to measure, meaning the circumstance in which a measure of a construct is positively related to another measure of the same construct.

The average variance shared between a concept and its measures is called the AVE. Indicators linked to a specific construct have their squared loadings calculated as the overall mean of squared loadings (the total squared loadings divided by the number of indicators), defined as such (62).

The average extracted variance (AVE) and item loadings are examined when analyzing the convergent validity of a measure using the PLS technique (62).

The average variance shared between a concept and its measures should be higher than the average variance shared between the construct and other latent variables in the same model, if possible (63).

Calculating AVE is included in the analytical program of the PLS environment. In this case, the concept explains more than half of the variation of its indicators, with an AVE value equal to or greater than 0.5. Conversely, an AVE lower than 0.50 suggests that there are more errors in the indicators than the average variance explained by the constructs, which is the case for most indicators. Therefore, an AVE value greater than or equal to 0.50 is considered acceptable in general (62).

Table 1 shows that all AVE values are above the acceptable threshold of 0.5, indicating that the convergent validity of the measurement model has been validated.

### 3.4 Discriminant validity

The discriminant validity of a concept concerns the uniqueness of the construct, meaning if the phenomenon claimed by a construct is distinct and not reflected by other latent variables in the model (62).

The Fornel-Larcker criterion tests the second discriminant validity of a concept by comparing the square root of AVE values with correlations of latent variables (60).

In Table 2, the discriminant validity of the Fornel-Larcker criteria and cross-loadings is examined, and the results show that discriminant validity is satisfactory.

**Table 2:** Fornell-Larcker criterion.

	Body appearance	Psychological distress	Physical activity	Marital satisfaction	Sexual self-esteem	Sedentary
Body appearance	0.834					
Psychological distress	−0.469	0.851				
Physical activity	−0.395	0.299	0.842			
Marital satisfaction	0.42	−0.539	−0.53	0.873		
Sexual self-esteem	0.536	−0.447	−0.604	0.608	0.901	
Sedentary	−0.328	0.23	0.759	−0.429	−0.486	1

An investigation by Henseler et al. (62), based on their Monte Carlo simulation, introduced a new criterion called HTMT that can be used to assess the effectiveness of a treatment. According to Henseler and colleagues,for discriminant validity to be achieved, the HTMT score must fall within the confidence interval values of −1 and 1.

Using the Fornell-Larcker criteria and the Heterotrait-Monotrait Ratio of correlations, discriminant validity can be determined by examining the cross-loading between the study components (HTMT). Therefore, for a valid discriminant construct, its loadings on itself should be high while its loadings on other constructs are low (see Table 3) (64).

**Table 3:** Heterotrait-Monotrait ratio.

	Body appearance	Psychological distress	Physical activity	Marital satisfaction	Sexual self-esteem
Psychological distress	0.487				
Physical activity	0.435	0.327			
Marital satisfaction	0.466	0.6	0.619		
Sexual self-esteem	0.564	0.471	0.668	0.681	
Sedentary	0.336	0.235	0.816	0.467	0.501

Along the diagonal of the correlation matrix, square roots of AVE coefficients are displayed. The square roots of AVEs should be higher than their greatest association with all other constructs to be considered significant (60).

### 3.5 Evaluation of the structural model

The structural model focuses on latent variables and indicates the theoretically established relationship that exists between the provided data on both the input and output sides of the equation. The purpose of the analysis, based on latent variables, is to predict output training data based on the input data in the model, using the data from latent constructs. In another formulation, the structural model is used to demonstrate one or more dependent relationships that are conceptually linked to the idea of the considered model. An evaluation technique for the structural model was presented by Hair and colleagues, which included several consecutive steps that could all be completed in a single session (60).

### 3.6 Evaluation of the structural model for multicollinearity

When two or more independent variables are highly associated with each other, multicollinearity is present in the data. Multicollinearity in ordinary least squares regression inflates standard errors, renders tests of variation of an independent variable inaccurate, and prevents the researcher from determining the relative importance of one independent variable over another (65).When the variance inflation factor (VIF) coefficient is greater than 4, multicollinearity might be an issue.

We found that the PLS method generated VIF values ranging from 1.919 for item APQ3 to 4.046 for item SS1, depending on the indicator we examined. With the exception of SS1, all numbers are below 4, demonstrating that the multicollinearity problem does not exist (see Table 4).

**Table 4:** Multicollinearity.

Items	VIF	Items	VIF
APQ1	1.989	SS1	4.046
APQ2	2.029	SS2	3.755
APQ3	1.919	SS3	2.851
APQ4	2.249	SS4	3.342
DAS1	2.162	SS5	3.812
DAS2	1.913	ac1	3.461
DAS3	2.019	ac10	2.486
K1	3.801	ac2	3.256
K2	3.474	ac3	2.775
K3	3.493	ac4	3.114
K4	3.406	ac5	3.015
K5	3.887	ac6	3.101
K6	3.366	ac7	2.661
K7	3.413	ac8	3.633
K8	2.959	ac9	3.292
K9	3.144		
K10	3.283		

AP, physical activity; DAS, the dyadic adjustment scale (DA S-4); K, Kessler scale (K10); SVS, sexual satisfaction; AC, body appearance.

### 3.7 Evaluation of *R*-squared and adjusted *R*-squared

Using the values of *R*-squared (*R*^2^) for endogenous variables, the predictive ability of a specific model or construct, as well as the derivation of the standard path coefficient for each connection between exogenous and endogenous variables, are assessed in PLS analysis. The significance of *R*^2^ values in SmartPLS is similar to interpreting *R*^2^ values obtained from multiple regression analysis. In the construct, *R*^2^ values reflect the extent to which the variation of the construct can be explained by the model. The degree of variation explained by the external variable in its endogenous counterpart is shown by the *R*^2^ statistic (66). It represents a depiction of the model's parameter quality (54). However, Adjusted *R*-squared is a modified form of *R*-squared in which the number of predictors in the model has been corrected. The updated *R*^2^ increases only if the additional term improves the model more than expected. It decreases when a predictor enhances the model by an amount lower than what would have been anticipated by chance.

According to Chin (66), an *R*^2^ of 0.67 is considered high, 0.33 is considered moderate, and 0.19 is considered low.

Psychological distress has an *R*^2^ value of 0.57, indicating that the combined effect of sedentary behavior and marital satisfaction can lead to a change in psychological distress by 22%, while the adjusted *R*^2^ value for psychological distress is 21.8%. Both *R*^2^ and adjusted *R*^2^ values show weak effects. Their complexity is determined by the model's complexity and research discipline (67).

Similarly, the combined impacts of AEC, SEL, and EVAL-CAUSE on company reputation represent 65.8% of the variance in this variable.

On the other hand, psychological distress, sexual life satisfaction, physical activity, and sedentary behavior managed to explain 49.6% of marital satisfaction. However, body appearance explained 28.7% of sexual life satisfaction (refer to Table 5).

**Table 5:** *R*-squared and adjusted *R*-squared.

Construits	*R* Square	*R* Square Adjusted
Psychological distress	0.22	0.218
Physical activity	0.575	0.574
Marital satisfaction	0.496	0.492
Sexual self-esteem	0.287	0.286

### 3.8 *F* square effect size

Cohen's *f*^2^ values have also been calculated to emphasize the significance of the pathway. *f*^2^ is a measure of the magnitude of an effect caused by a pathway.

*f*^2^ can be calculated using the following equation: *f*^2^ = *R*^2^ (included) - *R*^2^ (excluded)/1 - *R*^2^ (included).

To interpret the effect size, Cohen recommends using the following criteria: small effect size 0.02; medium effect size 0.15; and large effect size 0.35 (68).

The results are presented in Table 6.

**Table 6:** Effect size *f*^2^.

	Psychological distress	Physical activity	Marital satisfaction	Sexual self-esteem
Body appearance	0.282			0.403
Psychological distress			0.168	
Physical activity			0.032	
Sexual self-esteem			0.109	
Sedentary		1.355	0.001	

### 3.9 Evaluation of the predictive significance of a model *Q*^2^

*R*-squared values gauge the model's predictive accuracy, while the predictive index *Q*^2^ indicates the accuracy in prediction, often referred to as “Stone-Geisser's *T*^2^ value” (60, 66). A *Q*^2^ value above zero signifies the predictive relevance of the relationship for that specific construct (56, 60).

*Q*^2^ values can be determined using the Blindfolding process for an omission distance *D* that has been specified. “Blindfolding” is a resampling approach where each data point in endogenous construct indicators is omitted, and parameters are estimated using the remaining data points from the remaining data (62, 69).

Assessing the effect size of *Q*^2^ is important for determining whether exogenous constructs have a significant influence on the endogenous construct. It's crucial to evaluate both the significance of the pathway being considered and how much it changes the explanatory power of the endogenous concept (70). When used in isolation, the path coefficient doesn't provide any information about the significance of the influence of exogenous latent variables on endogenous constructs.

During the effect size estimation process (*f*^2^), it's advisable to consider the predictive relevance (*Q*^2^) of the model, which was done to determine its predictive ability.

The Stone-Geisser test, a non-parametric test, can be used to determine the predictive significance of the research design (66). As a result of the outcome extracted from cross-validation, it's possible to estimate the predictability of endogenous constructs and, consequently, the overall quality of the model. According to Hair et al. (57), *Q*^2^ not only analyzes model values but also estimates the values and parameters of the model in question.

For the SmartPLS model presented in this study, the *Q*^2^ effect sizes demonstrate the predictive relevance of coefficients, all of which are greater than 0 (see Table 7).

**Table 7:** *Q*^2^ effect sizes.

	SSO	SSE	*Q*^2^ (=1-SSE/SSO)
Body appearance	4,430	4,430	
Psychological distress	4,430	3,734.134	0.157
Physical activity	1,772	1,056.244	0.404
Marital satisfaction	1,329	834.165	0.372
Sexual self-esteem	2,215	1,702.613	0.231

### 3.10 Mean squared error (RMSE)

According to Henseler and Sarstedt (62), the Root Mean Squared Error (RMSE) can be used as a goodness-of-fit statistic in the PLS-SEM approach. It represents the difference between the observed correlation and the expected correlation expressed as a root mean squared error. Hence, the average size of the discrepancies between observed and anticipated correlations can be used to assess model fit criteria in an absolute sense. A good fit is defined as a result less than 0.10 and preferably less than 0.08 (60).

The model generated a Root Mean Squared Error (RMSE) with a reasonable value of 0.045, as indicated in Table 8.

**Table 8:** Root mean squared error (RMSE).

	Saturated model	Estimated model
SRMR	0.045	0.112
d_ULS	1.16	7.085
d_G	0.774	0.849
Chi-Square	1,936.056	2,037.04
NFI	0.858	0.851

### 3.11 Verification of direct and indirect link hypotheses

The execution of the PLS algorithm and bootstrapping calculations in the SmartPLS software provided the path coefficient of these relationships, indicating the strength of the relationships, along with the *P*-value, which can be used to determine if the relationship is statistically significant or not (refer to Table 9 and Table 10).

**Table 9:** Direct effects.

	Original sample (O)	Sample mean (M)	Standard deviation (STDEV)	*T* statistics (|O/STDEV|)	*P* values
Body appearance-> Psychological distress	−0.469	−0.467	0.05	9.383	0.0000
Body appearance-> Sexual self-esteem	0.536	0.535	0.032	16.989	0.0000
Psychological distress -> Marital satisfaction	−0.326	−0.328	0.04	8.067	0.0000
Physical activity -> Marital satisfaction	−0.213	−0.209	0.062	3.413	0.0010
Sexual self-esteem -> Marital satisfaction	0.315	0.316	0.05	6.256	0.0000
Sedentary -> Physical activity	0.759	0.759	0.023	33.491	0.0000
Sedentary -> Marital satisfaction	−0.039	−0.039	0.056	0.705	0.4810

**Table 10:** Indirect effects.

	Original sample (O)	Sample mean (M)	Standard deviation (STDEV)	*T* statistics (|O/STDEV|)	*P* values
Body appearance -> Marital satisfaction	0.321	0.322	0.033	9.771	0.000
Sedentary -> Marital satisfaction	−0.162	−0.159	0.048	3.337	0.001

For the direct relationships, except for the effect of sedentary behavior on marital satisfaction, all links exhibit highly significant effects.

For the indirect effects, significant relationships have been identified between physical appearance and marital satisfaction. Similarly, sedentary behavior showed an indirect effect on marital satisfaction.

## 4 Discussion

The results of this analysis, conducted through PLS modeling with SmartPLS, unveil a complex landscape of interactions among the variables under investigation. This meticulous exploration provides striking insights into the subtle relationships that shape our understanding of the dynamics at play.

Regarding reliability and validity, the high coefficients of Cronbach's alpha reliability, Rho-A composite reliability, and composite reliability indicate robust internal consistency of the measures. Moreover, AVE values exceeding 0.5 confirm convergent validity of the indicators, highlighting the measures' ability to capture the constructs they are intended to assess.

Discriminant validity is also satisfactory, as indicated by the Fornell-Larcker criterion and HTMT Ratio results, emphasizing the distinction between constructs and thereby reinforcing the measures' credibility.

Concerning the structural model, the *R*-squared (*R*^2^) and adjusted *R*-squared (*R*^2^ adjusted) values describe the model's ability to explain the variance of dependent variables. Marital satisfaction is influenced by factors such as distress, physical activity, and sexual life satisfaction. However, the relatively modest *R*^2^ values suggest that other variables not included in the model might also contribute to the variance.

High effect sizes (*f*^2^), particularly for body appearance, distress, and physical activity, underscore the substantial importance of these variables in the model. These significant effects suggest that changes in these variables can notably impact marital satisfaction.

Regarding predictability (*Q*^2^), positive values indicate predictive relevance of the model, thereby reinforcing the external validity of established relationships.

The low Root Mean Squared Error (RMSE) value indicates a reasonable fit of the model to the data, suggesting that the model is capable of faithfully reproducing relationships among the studied variables.

### 4.1 Discussion of hypotheses

The discussion of the hypotheses formulated in our study reveals significant results that shed light on the complex relationships between various psychological and relational factors (see Supplementary Figure 1). First, hypothesis H1 suggests a correlation between body self-esteem and sexual self-esteem. Our study confirms this hypothesis by establishing a link between body self-esteem and sexual satisfaction. Individuals with a positive body image demonstrated higher sexual satisfaction, which is consistent with previous research: For example, studies on body image suggest a correlation between body perception and sexuality. Thus, the literature highlights that among young women, a negative body image is often linked to sexual problems, sexual dysfunction, and sexual dissatisfaction (71).

Various authors claim that body image has an impact on sexual activity (72–74). Our findings align with the results of Hannier et al. (75), which highlight a relationship between women's sexual self-esteem and their satisfaction with their bodies., in a survey study published in a women's health and fitness magazine, women aged 14–74 reported that body image satisfaction was associated with greater comfort with their bodies during sexual activity, higher frequency of sexual behaviors, including increased initiation of sexual activity by women, and higher orgasm frequency (76). Similarly, among a non-clinical sample of 187 college female students, Hoyt and Kogan found that women dissatisfied with their sex lives and/or dating lives experienced more body image dissatisfaction compared to women satisfied with their sex lives (77). The authors suggested that these results could be attributed to discomfort in intimate situations involving female nudity, such as undressing in front of one's sexual partner, especially among women with low body self-esteem. Furthermore, studies on body image suggest a correlation between body perception and sexuality.

However, some studies have failed to establish this correlation. For instance, in two studies involving middle-aged women, no significant association was observed between body image and sexual satisfaction, especially after adjusting for other variables such as menopausal symptoms and mood (41).

Hypothesis H2, proposing a connection between sexual self-esteem and marital satisfaction, finds support in our findings. Our results suggest that individuals with positive sexual self-esteem tend to experience higher levels of marital satisfaction, which is consistent with prior research (78–81). Longitudinal studies have consistently reported that fluctuations in sexual satisfaction are closely linked to fluctuations in marital satisfaction (34, 39). Furthermore, a multitude of studies have demonstrated a positive association between sexual satisfaction and marital satisfaction (29–35).

Numerous studies have highlighted a significant and positive association between sexual satisfaction and the quality or satisfaction of the marital relationship (33, 82–84). Many investigations exploring the link between sexual and marital satisfaction have indicated that these two variables significantly predict each other (85–87). Brezsnyak, Fielder, and Klemer suggests that “A good sexual adjustment usually, but not always, requires a fairly good total marriage relationship” (85).

However, some researchers have suggested that there may not always be a direct relationship between marital satisfaction and sexual satisfaction (88, 89). Pazak and Berg-Cross propose that sexual dissatisfaction may occur even within otherwise happy marriages. Additionally, it's noted that sexually satisfied spouses may still experience dissatisfaction within their marriages. Similarly, Colebrook Seymour, found no significant relationship between marital satisfaction and sexual satisfaction (90).

Furthermore, our study confirms hypothesis H3, which highlights a correlation between marital satisfaction and body appearance. We observed a significant indirect effect between body appearance and marital satisfaction, suggesting that this influence is mediated by other factors not explicitly measured in our study. Individuals with higher marital satisfaction also exhibited a more positive perception of their body appearance, which reinforces previous findings (91–93).

Several studies support the notion that dissatisfaction with one's body is correlated with dissatisfaction in marital relationships, and vice versa, even after adjusting for variables such as age, body mass index, self-esteem, and gender (77, 94, 95). Specifically, women's bodydissatisfaction is associated with dissatisfaction in their marital relationships for both partners. This implies that not only are women who view themselves negatively less satisfied with their marital relationship, but their partners are also less satisfied (96). However, there is only one study that does not find a significant link between these variables (97).

In summary, our findings contribute to the growing body of literature suggesting a complex interplay between body image and marital satisfaction. While our study provides evidence of a correlation between marital satisfaction and body appearance, further research is needed to elucidate the underlying mechanisms and potential mediating factors in this relationship.

The validation of hypothesis H4, which suggests a correlation between psychological distress and marital satisfaction, is reinforced by our findings, consistent with existing research. Our results align with studies indicating that higher levels of psychological distress are significantly associated with lower marital satisfaction, as documented in previous literature (45, 98). Numerous studies have demonstrated that individuals facing heightened psychological distress tend to report lower quality in their marital relationships (42–44). Furthermore, our findings correspond with a study conducted by Karen (99), which highlights a negative association between marital satisfaction and psychological distress, emphasizing the importance of marital quality as a predictor of mental health status among married African Americans. This convergence of evidence underscores the significance of considering marital dynamics in comprehending psychological well-being.

Finally, our study supports hypothesis H5 positing a relationship between sedentary behavior and marital satisfaction. We observed a significant indirect effect of sedentary behavior on marital satisfaction, suggesting that this relationship is influenced by intermediary variables not accounted for in our initial model. Thus, no previous study had explicitly highlighted this link between sports and dyadic adjustment. In reality, what our results support is rather an indirect link, supported on the one hand by numerous theories linking sports practices to better psychological well-being (11), and on the other hand by studies associating low psychological distress with better marital satisfaction (42–44).

These findings offer valuable perspectives for targeted interventions aimed at improving relationship quality. However, it is essential to acknowledge the limitations of our study, particularly its cross-sectional nature, and to encourage future research to deepen the understanding of these complex relationships.

## 6 Conclusion

Firstly, our findings confirm the relationship between body self-esteem and sexual satisfaction, highlighting the importance of a positive body image in enhancing sexual well-being. This reaffirms the notion that individuals with higher levels of body satisfaction tend to experience greater satisfaction in their sexual relationships.

Secondly, we have established a link between sexual self-esteem and marital satisfaction, underscoring the role of positive sexual self-perception in fostering greater satisfaction within the marital relationship. This aligns with prior research emphasizing the interdependence of sexual and marital satisfaction.

Moreover, our study identifies a correlation between marital satisfaction and body appearance, indicating that individuals with higher levels of marital satisfaction also tend to perceive their body appearance more positively. This suggests a bidirectional relationship between body image and marital quality, warranting further exploration.

Additionally, our findings support the association between psychological distress and marital satisfaction, emphasizing the detrimental impact of psychological well-being on the quality of marital relationships. This underscores the importance of addressing psychological distress in interventions aimed at enhancing marital satisfaction.

Lastly, we have revealed an indirect relationship between sedentary behavior and marital satisfaction, suggesting that engagement in physical activities may indirectly influence relationship quality through its effects on psychological well-being. This highlights the potential role of lifestyle factors in shaping marital dynamics.

### 6.1 Limitations

Despite the valuable insights provided by our study, several limitations must be acknowledged. Firstly, the cross-sectional nature of our research design limits our ability to establish causal relationships between the variables studied. Future longitudinal studies are warranted to elucidate the temporal dynamics of these associations.

Secondly, our reliance on self-report measures introduces the potential for response bias and social desirability effects, which may influence the accuracy of our findings. Incorporating objective measures and observational data could enhance the validity of future investigations.

Furthermore, our study focused primarily on heterosexual couples, limiting the generalizability of our findings to diverse populations. Future research should strive to include more diverse samples to ensure the applicability of our findings across different demographic groups.

Moreover, our study did not explore the influence of cultural and societal factors on the variables under investigation. Cultural differences in attitudes towards sexuality, body image, and marital dynamics may significantly impact the observed associations. Therefore, future studies should consider cultural context in their analyses.

In conclusion, while our study contributes to a deeper understanding of the complex interplay between psychological, relational, and behavioral factors in marital satisfaction, further research is needed to address the limitations and provide more comprehensive insights into this important area of inquiry.

## Data Availability

The original contributions presented in the study are included in the article/Supplementary Material, further inquiries can be directed to the corresponding author.

## Supplementary material

Supplementary Figure 1 Model SMARTPLS.
