# Supplementary figures and images for: Balance and deep connections: the impact of physical activity on body and sexual self-esteem, psychological distress, and marital satisfaction among adults
Source: Front Sports Act Living. 2024 Apr 11;6:1343951. doi: 10.3389/fspor.2024.1343951 (PMC11043539; doi:10.3389/fspor.2024.1343951)

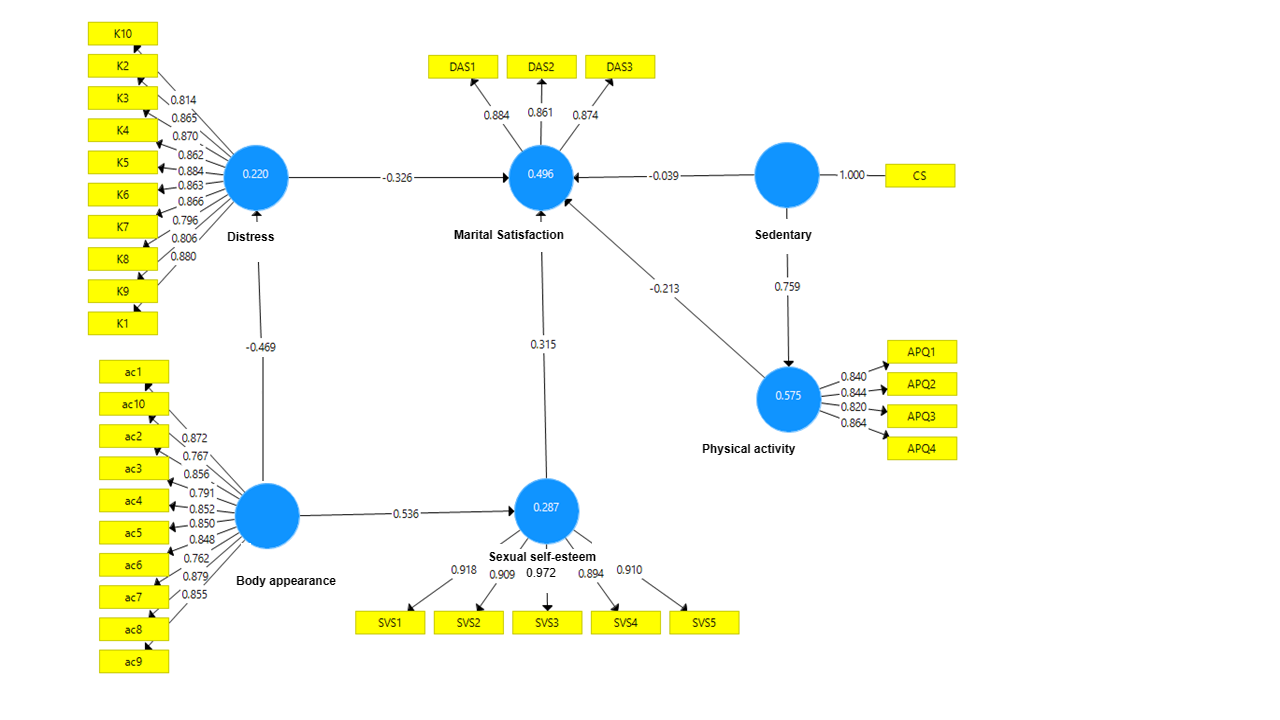

Supplement: Supplementary Figure 1 — Model SMARTPLS. [file Image1.png]
